# Involvement of the endogenous retroviral locus HERV-Fc1 on the human X-chromosome in multiple sclerosis

**DOI:** 10.1186/1742-4690-8-S2-P54

**Published:** 2011-10-03

**Authors:** Bjørn A Nexø, Bettina Hansen, Magdalena J Laska, Annette B Oturai, Helle B Søndergaard, Hanne F Harbo, Elisabeth G Celius, Karl K Nissen, Thor Petersen

**Affiliations:** 1 Department of Biomedicine, Aarhus University, DK-8000 Aarhus C, Denmark; 2 Danish Multiple Sclerosis Center, Department of Neurology, Copenhagen University Hospital Rigshospitalet, DK-2100 Copenhagen, Denmark; 3 Department of Neurology, Oslo University Hospital, Ullevål N-0424 Oslo, Norway; 4 Institute of Clinical Medicine, University of Oslo, Norway; 5 Department of Neurology, Aarhus University Hospital, 8000 Aarhus C, Denmark

## Background

Multiple sclerosis (MS) is considered a multifactorial disease with both genetic and environmental components in the etiology. Certain demyelinating diseases in other species have been shown to be caused by retroviruses, and the possibility remains that also MS has a viral component. As there is little evidence that MS is contagious, we have focused our attention on the human endogenous retroviruses, which are transmitted vertically from parent to offspring as Mendelian loci.

## Materials and methods

Forty-eight near-intact human endogenous retroviral loci were analyzed for association with MS by genetic epidemiology [1]. Four cohorts with total of 1740 DNAs from patients with definite MS living in Denmark (n = 1103) and Norway (n = 637), as well as 2841 controls living in the same parts of the countries were analyzed on a Sequenom facility.

## Results

The marker rs391745, located next to the HERV-Fc1 locus, was found to be associated with MS susceptibility in three of the four cohorts (corrected combined p-value 0.0001). Also, quantification of HERV-Fc1 RNA expression in plasma indicated that the content of RNA from this virus was four fold higher in patients with recent attacks relative to patients in stable remission and healthy controls (p =0.001) (See abstract by Laska MJ et al). Finally, the association of the marker rs391745 with subtypes of MS was further investigated in a subgroup of 1157 cases and 1886 controls among the patients mentioned above (Danish and Norwegian). Bout onset MS, comprising remitting/relapsing MS and secondary progressive MS, showed association with rs391745 (p = 0.003), while primary progressive MS did not (p = 0.96). (For molecular investigations of HERV-Fc1 see abstract by Nissen KK et al).

## Conclusions

The association of the HERV-Fc1 locus with MS and the ability to see genetic differences between subtypes of MS both speak for the involvement of HERV-Fc1 in some forms of multiple sclerosis. The heightened expression of HERV-Fc1 in relation to attacks may give clues to further studies of the pathogenesis.
